# Efficacy and Safety of Laser Therapy on Ischemic Central Retinal Vein Occlusion: A Systematic Review and Analysis of Clinical Studies

**DOI:** 10.7759/cureus.62292

**Published:** 2024-06-13

**Authors:** Ghadi F Alotaibi, Hadeel Seraj, Qasem A AlMulihi, Amnah A Alkhawajah, Salman G Eshbeer, Arwa A Alghamdi, Arwa M AlTowairqi, Shahad S Aloufi, Azizah M Alshubayni

**Affiliations:** 1 Faculty of Medicine, Taif University, Taif, SAU; 2 Ophthalmology, King Abdulaziz University, Jeddah, SAU; 3 Emergency, Ministry of Health Holdings, Al Khobar, SAU; 4 Medicine and Surgery, King Faisal University, Al Ahsa, SAU; 5 Medicine and Surgery, Ibn Sina National College for Medical Studies, Jeddah, SAU; 6 Medicine, Princess Nourah Bint Abdulrahman University, Riyadh, SAU; 7 Medicine and Surgery, Taif University, Taif, SAU; 8 Medicine and Surgery, Taibah University, Madinah, SAU; 9 General Practice, King Faisal Specialist Hospital & Research Centre, Tabuk, SAU

**Keywords:** macular edema, panretinal photocoagulation, laser, crvo, ischemic central retinal vein occlusion

## Abstract

Many studies have evaluated different treatments for ischemic central retinal vein occlusion (CRVO). Nevertheless, improvement and complication rates vary significantly. This systematic review aimed to evaluate the efficacy of laser therapy in treating ischemic CRVO compared with a control group using other treatments. The databases of PubMed, Google Scholar, and ClinicalTrials.gov were searched using a variety of keywords, including “ischemic central retinal vein occlusion,” “CRVO,” “laser,” and “panretinal photocoagulation.” After data extraction, each study’s quality was assessed using the methodological index for nonrandomized studies (MINORS) or grading of recommendations, assessment, development, and evaluation or GRADE standards. A sum of 195 abstracts were reviewed, and seven clinical trials were eventually chosen. Of these, four were prospective studies, two were randomized controlled studies, and only one was a retrospective study. The assessment of potential biases in our included studies revealed that all these studies demonstrated moderate or high quality. Two studies were selected for meta-analysis, and the results showed no significant difference in visual acuity (VA) outcomes between the treated and the control groups (P = 0.17). In the remaining five studies, laser therapy was found to be more effective at neovascular complications, with a higher rate of neovascular glaucoma (NVG), iris neovascularization (NVI), neovascularisation at disc (NVD), and retinal neovascularization in the group without laser treatments. This review suggests that laser therapy is essential in preventing neovascular complications, such as NVG, NVI, NVD, and retinal neovascularization rather than improving VA. In addition, the combination of laser photocoagulation and intravitreal injection (IVI) improved VA, but further studies are required.

## Introduction and background

Ischemic central retinal vein occlusion (CRVO) represents a formidable challenge in ophthalmology, attributed to its potential for inducing severe vision loss through the obstruction of the central retinal vein. This condition leads to retinal swelling and damage, culminating in significant visual impairment. The demographic distribution of CRVO incidence highlights a preference for males, with a notable increase in prevalence correlating with advancing age. A diverse array of risk factors, including but not limited to aging, cardiovascular disease, diabetes, hypertension, and lifestyle factors such as smoking and obesity, underscores the complexity of its etiology. Moreover, CRVO can precipitate neovascular glaucoma (NVG), a devastating complication characterized by painful red eyes and decreased visual acuity due to elevated intraocular pressure, further complicating the clinical management of affected individuals [1-4].

Within the therapeutic spectrum, laser therapy has been a cornerstone in managing both ischemic and nonischemic CRVO, primarily through procedures such as laser photocoagulation aimed at reducing macular edema and preventing neovascularisation. Despite its utility, the efficacy of laser photocoagulation in improving visual acuity in CRVO has been limited, contrasting its more pronounced benefits in cases of branch retinal vein occlusion (BRVO) [5,6]. The advent of pharmacological interventions, notably anti-VEGF agents, has heralded a paradigm shift in treatment approaches, offering new avenues for addressing the underlying pathophysiological mechanisms of CRVO [7-9].

The evolution of treatment strategies from laser photocoagulation to include anti-VEGF therapies reflects a nuanced understanding of CRVO's multifactorial nature. Recent investigations into combination therapies, such as ranibizumab with laser photocoagulation, underscore an ongoing quest to optimize therapeutic outcomes by enhancing macular volume and best-corrected visual acuity [7]. This systematic review seeks to elucidate laser therapy's comparative effectiveness and safety against a backdrop of rapidly evolving pharmacologic treatments, with a particular focus on anti-VEGF modalities. By thoroughly examining clinical trials assessing laser therapy's role in managing ischemic CRVO, this analysis aims to synthesize existing evidence and delineate optimal treatment strategies.

Emphasizing methodological rigor, this study incorporates a comprehensive assessment of research studies exploring laser therapy applications in adults with ischemic CRVO. The systematic review analyzes findings from various clinical trials to ascertain laser therapy's overall efficacy and safety juxtaposed with alternative treatment options. This endeavor is poised to contribute significantly to the clinical management of ischemic CRVO, guiding healthcare providers and patients through the complexities of treatment selection in an era marked by therapeutic innovation.

## Review

Methods

Search Strategy

This systematic review was prospectively enrolled in PROSPERO (CRD42023458211) and carried out by the guidelines for the preferred reporting items for systematic reviews and meta-analyses (PRISMA). We thoroughly searched the following electronic databases: ClinicalTrials.gov, online trials registers, Medline, Embase, Google Scholar, PubMed, and the Cochrane Central Register of Controlled Trials (CENTRAL). The search strategy was independently developed by one of the authors (G.F.): (ischemic occlusion of the central retinal veins OR CRVO) AND (laser OR panretinal photocoagulation OR PRP) AND (intravitreal anti-VEGF OR anti-VEGF OR corticosteroid therapy) AND (Macular edema OR neovascular glaucoma OR NVG OR ocular neovascularisation OR ocular NV OR iris neovascularisation OR NVI) AND (visual loss OR visual deterioration OR visual impairment).

Inclusion/Exclusion Criteria

English language studies that assessed how laser treatment affected ischemic central retinal vein blockage and provided data on patients over 18 and those published without time restrictions were included in the review. Studies that provided results of interest pertinent to the clinical issues were those in which full medical data were accessible for at least six months after therapy. The review also covered prospective cohort studies and randomized controlled trials (RCTs). All research (such as editorials, letters, comments, or reviews) that were not prospective cohort studies or RCTs and studies that did not report results of interest for the clinical issues and that involved children or patients under the age of 18 were excluded. Laser therapy-free studies were not included. Depending on how the study design, sample size, data collection and analysis, and other pertinent aspects were evaluated, some studies had a significant risk of bias or were of low quality. Studies that were determined to be seriously biased were also eliminated.

Data Extraction

Author, publication year, country, sample size, study design, duration of follow-up, types of treatments, and inclusion/exclusion criteria were among the study characteristics retrieved from the preserved studies. Age, sex, medical history, medications, and diagnosis are all features of the patient. Following up procedures and treatment protocols are intervention characteristics. VA, complications, and quality of life are outcome measures. Data analysis covered the statistical methods used, the effect size, the confidence intervals, the heterogeneity, and the publication bias. The conclusion covered key findings, restrictions, and suggestions for further investigation.

Bias Assessment

We used a Cochrane risk of bias tool to apply the methodological index for nonrandomized studies (MINORS) for nonrandomized comparative studies and a revised Cochrane risk of bias tool (RoB2) for RCTs. Every research category was assessed for inadequate data, biased reporting, blinding of observers, randomization, allocation concealment, and staff. Based on these evaluations, each category was rated as “low risk,” “high risk,” or “some concern.”

The included nonrandomized trials were evaluated using the MINORS tool. This tool assesses the quality of nonrandomized studies based on 12 items, each scored from 0 to 2. A score of 0 indicates that the item was not reported, 1 indicates that the item was inadequately reported, and 2 indicates that the item was adequately reported. The total score ranges from 0 to 24, with higher scores indicating better study quality.

Results

Search Results

**Figure 1:**
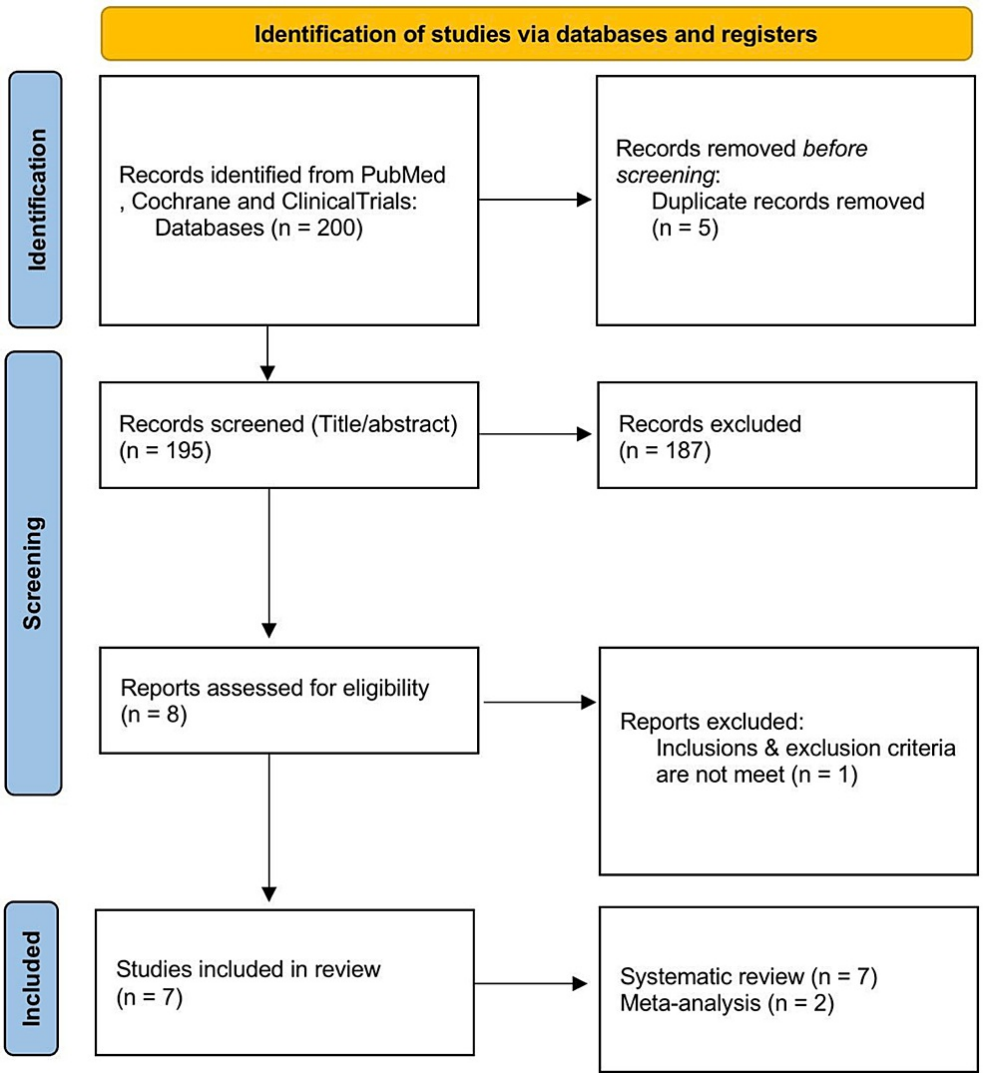
PRISMA flow chart Preferred reporting items for systematic reviews and meta-analyses (PRISMA)

The search yielded 200 articles (94 from PubMed, 41 from Cochrane, and 60 from ClinicalTrials.gov), and 192 of them (including duplicates) were excluded after reviewing their titles and abstracts for several reasons. First, many studies did not focus on laser treatment for ischemic CRVO, rendering them irrelevant. Second, some studies included patients under the age of 18 or involved animal models, which did not meet the inclusion criteria. Third, certain publications were editorial pieces, letters, comments, or reviews, rather than prospective cohort studies or randomized controlled trials (RCTs). Fourth, several studies did not provide results pertinent to the clinical issues, such as efficacy and safety outcomes. Finally, some studies lacked at least six months of follow-up data post-therapy, disqualifying them from inclusion. An additional article was eliminated following a full-text review. Seven studies were ultimately included in the current systematic evaluation (Figure 1). Three studies evaluated the effectiveness of laser treatments compared with alternative treatments (with or without drugs) for ischemic CRVO. In addition, three studies assessed the efficacy of early laser therapy compared with a group that received no early treatment. The remaining study evaluated the effectiveness of panretinal photocoagulation (PRP) therapy plus ranibizumab injections for ischemic CRVO. Table 1 displays the details of the included studies. Out of the nine remaining studies, seven were ultimately included in the current systematic evaluation, and two were selected for meta-analysis. The inclusion of these two studies in the meta-analysis was due to their robust design, comprehensive data, and relevance to the research question. These studies provided detailed quantitative data on the efficacy and safety of laser treatment for ischemic CRVO, allowing for a rigorous statistical comparison. Additionally, both studies had a follow-up period of at least six months and reported key clinical outcomes, such as VA and complication rates, which are critical for a meaningful meta-analysis. Their high methodological quality and low risk of bias further justified their inclusion, ensuring that the meta-analysis would yield reliable and generalizable results.

Features of the Included Research

Table 1 shows identified seven studies, comprising two RCTs [10,11] and five cohort studies [12-16], involving a cumulative patient count of 513. These studies span several countries, specifically the United States, Korea, China, and the United Kingdom (London), offering a diverse international perspective on the condition's treatment approaches.

**Table 1:** Characteristics of the studies included Central retinal vein occlusion (CRVO), panretinal photocoagulation (PRP), Radial optic neurotomy (RON), arteriovenous transit time (AVTT), neovascular glaucoma (NVG), Two Clock Hours of Iris Neovascularization or any Angle Neovascularization (TC-INV/ANV), iris neovascularization (INV)

First author (year) and type of study	Country	Diagnostic information	Patients (n)	Male/female (n)	Treatment in study group vs control group	Result	Follow-up after treatment (mean)
Hayreh et al. (1990), prospective cohort study [12]	USA	Ischemic CRVO	123 (laser group, 47; non-laser, 76)	Laser group, 27: 20; non-laser, 38: 38	Argon laser vs no treatment group	Early laser treatment after CRVO reduces iris neovascularization, with no other significant differences.	Every 6 months for 120 months
Kim et al. (2005), retrospective cohort study [13]	Korea	Ischemic CRVO	27 (PRP group, 16; RON, 11)	PRP, 5: 11. RON group, 5: 6	PRP vs RON group	No significant difference in visual acuity or AVTT between PRP and RON groups post-operation.	6-30 months
Magargal et al. (1981), prospective cohort study [14]	USA	Ischemic CRVO	100	N/A	PRP	Early argon laser PRP prevents NVG in high-risk ischemic CRVO eyes, with 93% of NVG cases having an ischemic index over 50%.	6-60 months
The Central Vein Occlusion Study (1995), RCT [11]	USA	Ischemic CRVO	181 (early group, 90; no early, 91)	84: 96	PRP	Prophylactic treatment didn't significantly reduce TC-INV/ANV development, with prompt regression observed in 56% of untreated and 22% of treated eyes. TC-INV/ANV was linked to non perfused retina and retinal hemorrhage severity, with higher risk in males and recent occlusions.	12-36 months
Zikui et al. (2004), prospective cohort study [15]	China	Ischemic CRVO	24	17:7	PRP	INV regressed after PRP with no significant change in visual acuity, no NVG, and two cases of vitreous hemorrhage.	3-24 months
Laatikainen et al. (1977), RCT [10]	London	Ischemic CRVO	48 (treatment group, 24; control, 24)	Intervention, 12: 12; control, 11:13	PRP	Photocoagulation improved neovascularization in ischemic CRVO without enhancing visual acuity, ineffective for hyperpermeability CRVO.	12 months
Spaide (2013), prospective cohort study [16]	N/A	Ischemic CRVO	10	N/A	PRP with ranibizumab	Laser treatment with 1,757 spots did not significantly change injection frequency or visual acuity in the study.	6 months

The RCTs and cohort studies present a detailed landscape of therapeutic outcomes for CRVO, with follow-up durations ranging from three to 120 months. This extensive range of follow-up times across studies from different geographical regions provides a comprehensive view of short-term and long-term treatment efficacies and patient management strategies in the context of ischemic CRVO. 

Bias Risk Assessment

**Figure 2:**
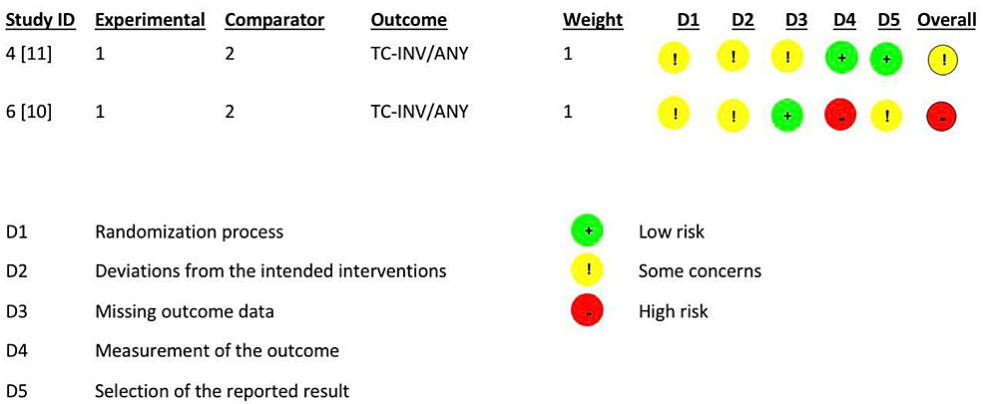
Cochrane risk of bias (RoB2) assessment tool for randomized trials

To evaluate bias risk, two researchers worked together and independently used the RoB2 tool for eligible RCTs. The revised Cochrane tool was employed, and according to this tool, one of the included RCTs was deemed to have a low risk of bias. At the same time, the other RCT raised some concerns regarding randomization, protocol deviations, and missing outcome data (Figure 2).

The included nonrandomized trials were evaluated using the MINORS tool [17]. The total MINORS score ranged from 13 to 20, with a mean score of 15.2 (Tables 2, 3). The lowest scores were in the elements of the prospective calculation of the study size (score of 0 or 1 in most studies, indicating inadequate or absent reporting) and the impartial evaluation of the endpoint (score of 0 or 1 in most studies, indicating inadequate or absent reporting). The highest scores were in the clarity of the aim of the study, the prospective collection of data, the appropriateness of the follow-up period to the aim of the study, and the clarity of endpoints (score of 2 in all studies, indicating adequate reporting).

**Table 2:** MINORS assessment tool for nonrandomized comparative studies Methodological index for nonrandomized studies (MINORS)

Item		
A clearly stated aim	2	2
Inclusion of consecutive patients	0	2
Prospective collection of data	2	2
Endpoints appropriate to the aim of the study	2	2
Unbiased assessment of the study endpoint	0	1
Follow-up period appropriate to the aim of the study	2	2
Loss to follow-up less than 5%	0	2
Prospective calculation of the study size	0	0
An adequate control group	1	1
Contemporary groups	2	2
Baseline equivalence of groups	2	2
Adequate statistical analyses	2	2
Total score	15	20

**Table 3:** MINORS assessment tool for nonrandomized non-comparative studies Methodological index for nonrandomized studies (MINORS)

Item			
A clearly stated aim	2	2	2
Inclusion of consecutive patients	2	2	2
Prospective collection of data	2	2	2
Endpoints appropriate to the aim of the study	2	2	2
Unbiased assessment of the study endpoint	2	1	0
Follow-up period appropriate to the aim of the study	2	2	2
Loss to follow-up less than 5%	1	2	2
Prospective calculation of the study size	0	2	1
Total score	13	15	13

Result of Qualitative analysis (Systemic Review)

In evaluating laser therapy for CRVO, studies reveal nuanced insights into efficacy and safety, with some variability in findings. The Central Vein Occlusion Study (CVOS) Group's RCT found that immediate PRP did not significantly prevent iris or angle neovascularisation, suggesting a more prudent approach of observation and timely PRP upon neovascularization development [11]. Similarly, Laatikainen et al.'s RCT indicated PRP's limited impact on VA improvement across CRVO types, recommending its use mainly for preventing complications in ischemic CRVO [10]. Conversely, some cohort studies, such as Hayreh et al.'s prospective study, showed that early PRP treatment reduced iris neovascularisation without major complication differences between treated and untreated groups [12]. Magargal et al. and Yu et al.’s prospective studies supported PRP's potential to prevent NVG and reduce iris neovascularization complications [14,15]. However, Kim et al.'s retrospective study and Spaide's prospective study highlighted PRP's limited efficacy in improving VA or reducing treatment frequency for CRVO, though they noted its safety profile [13,16]. These findings underline the safety of PRP while revealing its conditional efficacy in preventing neovascular complications in ischemic CRVO. The differing results among studies suggest that PRP's effectiveness may depend on individual patient risk profiles and the timing of intervention. Therefore, while PRP is generally safe, its application should be tailored to the specific needs of patients, considering both the potential benefits and the limitations reported in various studies.

Result of Quantitative Analysis (Meta-Analysis)

The data were processed and statistically analyzed using RevMan 5.2.3 software (Cochrane Collaboration, London, UK). To ensure accuracy, the data were double-checked before analysis began. The effect model utilized in the analysis relies upon the degree of study heterogeneity. P ≤ 0.05 indicates statistical significance in the difference between the two groups. A forest map is used to present the analysis's findings.

Two articles were selected for meta-analysis in CRVO patients (argon laser PRP in ischemic CRVO, a 10-year prospective study) and (comparative evaluation of radial optic neurotomy (RON) and PRP in the management of central retinal vein occlusion a retrospective uncontrolled study). We also assessed the effectiveness and safety of each treatment method by the development of NVG as a serious complication happens only with ischemic CRVO [12,13].

The first article compared laser and non-laser groups. This study included a well-defined control group where the non-lasered eyes were followed in parallel with the lasered eyes. The baseline characteristics between the laser and non-laser groups showed no statistically significant differences, indicating that the groups were comparable at the start of the study. This study's data demonstrated no statistically significant differences in the incidence of angle neovascularization (NV), NVG, retinal and/or optic disc NV, vitreous hemorrhage, or VA between the laser and non-laser groups, supporting the internal validity of the comparisons made​.

The second article compared laser therapy with other treatments, specifically RON. This study provided detailed baseline demographic data, showing no significant differences between the groups regarding age, presence of systemic diseases, or initial VA, thus establishing the comparability of the groups. VA and arteriovenous transit time (AVTT) changes over six months were assessed, with results indicating no significant differences between the PRP and RON groups. Furthermore, the incidence of NVG was reported, with NVG developing in two eyes that underwent PRP but not in any eyes from the RON group, further supporting the comparability and relevance of the control group in this context​.

**Figure 3:**
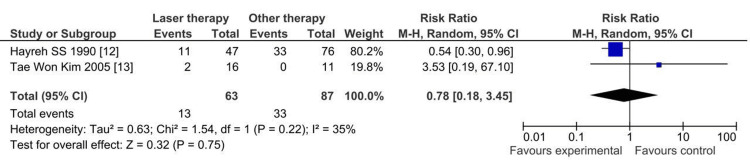
Comparison between the combination IVI and laser photocoagulation treatment group and the single IVI treatment group regarding NVG development Ref. [12,13] Neovascular glaucoma (NVG)

By ensuring that both studies had comparable control groups and provided comprehensive follow-up data, the meta-analysis could reliably assess the relative efficacy and safety of the different treatment methods. The p-value obtained from the meta-analysis indicated no significant difference (P = 0.75) between the treatment methods in preventing the development of NVG, suggesting comparable efficacy and safety profiles for the treatments evaluated (Figure 3).

Discussion 

CRVO is a potentially blinding condition caused by a blockage of the central retinal vein. Laser therapy, particularly PRP, is a treatment option for ischemic CRVO, aiming to alleviate complications by creating small burns in the retina to reduce ischemia and prevent neovascularization. The primary goal of our analysis was to evaluate the impact of laser therapy on ischemic central vein occlusion, assessing its efficacy by comparing outcomes between groups that received PRP and control groups that received other therapies or no treatment.

The CVOS Group conducted a randomized clinical trial involving 181 eyes, comparing immediate PRP in 90 eyes with observation and subsequent PRP upon the development of neovascularisation in 91 eyes. They found that, while immediate PRP did not significantly reduce the incidence of iris or angle neovascularisation, the observation strategy and timely intervention upon neovascularisation proved effective, suggesting an individualized approach to PRP application [11]. Hayreh et al. reported on a 10-year prospective study involving 123 eyes, observing no statistically significant difference in major complications between the laser-treated and non-treated groups. However, they noted a reduction in iris neovascularisation in the treated group when PRP was performed within 90 days of CRVO onset (P = 0.04), despite a significant peripheral visual field loss (P < 0.03), indicating a potential benefit of early PRP intervention for specific complications [12]. Magargal et al.'s prospective study emphasized PRP's preventive capability against NVG, with none of the treated eyes developing NVG unless exposed to another ischemic event, highlighting the potential of PRP to substantially mitigate the risk of NVG in high-risk ischemic CRVO eyes [14]. Kim et al. assessed PRP in 27 patients, revealing no statistically significant improvement in VA or AVTT post-treatment, highlighting the procedure's safety but limited efficacy in altering the clinical course of CRVO [13]. Yu et al.’s study involved 24 patients undergoing early PRP, showing regression of iris neovascularisation in all cases, with no significant change in VA and no NVG occurrences, underscoring early PRP's safety and efficacy in preventing specific ischemic CRVO complications [15]. Laatikainen et al. conducted an RCT comparing PRP's efficacy in CRVO patients, finding no significant improvement in VA. However, PRP reduced iris, disc, and retinal neovascularisation in the ischemic CRVO group, suggesting its usefulness in preventing complications rather than improving visual outcomes [10]. Spaide's prospective study on PRP and ranibizumab treatment found no significant difference in the frequency of ranibizumab injections or VA before and after PRP (injection frequency before PRP was 3.4 and 3.1 after PRP, P = 0.26; VA was 54.2 letters at the time of PRP and 51.4 letters at the end, P = 0.33), indicating PRP's limited additional benefit in pharmacologically managed CRVO patients [16].

The studies selected for meta-analysis showed that adding laser photocoagulation to IVI improved the best-corrected VA (BCVA). However, the improvement was nonsignificant compared with the non-laser group, which only received IVI treatment. While this systematic review provides insightful findings regarding the effectiveness of PRP in ischemic CRVO, several limitations must be noted: (1) Due to the lack of sufficient relevant studies, the meta-analysis conducted to compare BCVA among patients who had a combination treatment group IVI and laser photocoagulation and a single IVI treatment group included the results of only two studies. (2) Selection bias was unavoidable, as we included only studies published in English. Therefore, we recommend that future research efforts focus on conducting larger, multi-center RCTs with standardized treatment protocols and outcome measures to provide more definitive evidence on the efficacy and safety of PRP in ischemic CRVO.

## Conclusions

This systematic review provides a comprehensive view of the efficacy of laser therapy in treating central retinal vein occlusion and tries to determine the effectiveness of laser therapy compared with other treatment strategies. It is obvious that laser therapy, mainly PRP, is good for preventing neovascular complications, but surprisingly, it did not show any improvement in VA in patients with CRVO. Other treatment methods did not show a clear efficacy, but the combination of laser photocoagulation and IVI for improving VA is an area that warrants further investigation. This review suggests that laser therapy is effective in preventing neovascular complications such as NVG, NVI, NVD, and retinal neovascularisation rather than in improving VA. In addition, the combination of laser photocoagulation and IVI did not improve VA, but further studies are required.
